# Fitness, Fatness, and Mortality in Men and Women From the UK Biobank: Prospective Cohort Study

**DOI:** 10.1161/JAHA.120.019605

**Published:** 2021-03-13

**Authors:** Jakob Tarp, Anders Grøntved, Miguel A. Sanchez‐Lastra, Knut Eirik Dalene, Ding Ding, Ulf Ekelund

**Affiliations:** ^1^ Department of Sports Medicine Norwegian School of Sports Sciences Oslo Norway; ^2^ Research Unit for Exercise Epidemiology Centre of Research in Childhood Health Department of Sports Science and Clinical Biomechanics University of Southern Denmark Odense Denmark; ^3^ Department of Special Didactics Faculty of Educational Sciences and Sports University of Vigo Pontevedra Spain; ^4^ Prevention Research Collaboration Sydney School of Public Health The University of Sydney Camperdown New South Wales Australia; ^5^ Department of Chronic Diseases and Ageing Norwegian Institute of Public Health Oslo Norway

**Keywords:** adiposity, epidemiology, obesity, physical activity, physical exercise, Epidemiology, Lifestyle, Exercise, Obesity, Primary Prevention

## Abstract

**Background:**

Cardiorespiratory fitness may moderate the association between obesity and all‐cause mortality (ie, the “fat‐but‐fit” hypothesis), but unaddressed sources of bias are a concern.

**Methods and Results:**

Cardiorespiratory fitness was estimated as watts per kilogram from a submaximal bicycle test in 77 169 men and women from the UK Biobank cohort and combined with World Health Organization standard body mass index categories, yielding 9 unique fitness‐fatness combinations. We also formed fitness‐fatness combinations based on bioimpedance as a direct measure of body composition. All‐cause mortality was ascertained from death registries. Multivariable‐adjusted Cox regression models were used to estimate hazard ratios and 95% CIs. We examined the association between fitness‐fatness combinations and all‐cause mortality in models with progressively more conservative approaches for accounting for reverse causation, misclassification of body composition, and confounding. Over a median follow‐up of 7.7 years, 1731 participants died. In our base model, unfit men and women had higher risk of premature mortality irrespective of levels of adiposity, compared with the normal weight–fit reference. This pattern was attenuated but maintained with more conservative approaches in men, but not in women. In analysis stratified by sex and excluding individuals with prevalent major chronic disease and short follow‐up and using direct measures of body composition, mortality risk was 1.78 (95% CI, 1.17–2.71) times higher in unfit‐obese men but not higher in obese‐fit men (0.94 [95% CI, 0.60–1.48]). In contrast, there was no increased risk in obese‐unfit women (1.09 [95% CI, 0.44–1.05]) as compared with the reference.

**Conclusions:**

Cardiorespiratory fitness modified the association between obesity and mortality in men, but this pattern appeared susceptible to biases in women.

**Nonstandard Abbreviations and Acronyms**
ACLS: Aerobics Center Longitudinal Study
BALL ST: Ball State Adult Fitness Longitudinal Lifestyle Study
BF%: body fat percentage
CRF: cardiorespiratory fitness
FFM: fat‐free mass
VETS: Veterans Exercise Testing Study

**Clinical Perspective**

**What Is New?**

- Cardiorespiratory fitness may modify the association between obesity and premature mortality (the "fat‐but‐fit" hypothesis), but previous studies appear susceptible to biases and little is known about this relationship in women.
- Among 77 169 middle‐aged men and women from the UK Biobank prospective cohort study, there was some evidence of bias in the fat‐but‐fit hypothesis, but low cardiorespiratory fitness remained associated with higher mortality in all strata of weight status among men.
- Obesity was not associated with higher mortality in the most fit 40% of male participants; evidence supporting the fat‐but‐fit hypothesis was much less clear in women.

**What Are the Clinical Implications?**

- Clinicians should support their patients in performing regular physical activity of sufficient intensity to improve cardiorespiratory fitness irrespective of weight status.
- Obese men with high cardiorespiratory fitness are not at an increased risk of premature mortality.

Obesity and low cardiorespiratory fitness (CRF) are well‐established major predictors of premature mortality.^1^, ^2^, ^3^ It was 30 years ago that the first study found that CRF modifies the risk of premature mortality associated with obesity (defined by body mass index [BMI]), such that, compared with normal‐weight individuals with high CRF, the risk of mortality was only higher in those who were obese and unfit, but not among those who were obese and fit.^4^ A meta‐analysis^5^ attempted to synthesize the growing number of epidemiological studies on this "fat‐but‐fit" hypothesis,^6^, ^7^, ^8^, ^9^, ^10^, ^11^, ^12^, ^13^, ^14^, ^15^ and reached the same conclusion that CRF modified the association between obesity and mortality.

There are several sources of bias that reduce certainty in previous conclusions on the fat‐but‐fit hypothesis. The majority of studies included in the meta‐analysis^5^ are different subsamples from the ACLS (Aerobics Center Longitudinal Study),^6^, ^7^, ^8^, ^9^, ^10^, ^11^, ^12^, ^13^ which has led to inflated precision and limited generalizability of the findings.^16^ Further, 5 of 10 studies were restricted to individuals with specific disease conditions such as coronary artery disease^14^ or diabetes mellitus,^12^ which may introduce selection bias through collider stratification.^17^ Misclassification of fitness‐fatness combinations is another serious concern. BMI is a practical but imperfect measure of body fat with considerable misclassification of adiposity status,^18^ which may be differential with respect to fitness status because of differences in fat‐free mass (FFM). In addition, expressing CRF as a ratio to body weight does not remove the confounding effect of body weight.^19^, ^20^ Furthermore, weight loss caused by subclinical or clinical conditions are additional challenges for determining BMI‐associated mortality,^21^ usually resulting in a shift of the optimal BMI range towards the overweight or obese categories.^3^, ^21^, ^22^ Subclinical or clinical disease may also result in impaired functional capacity, potentially inflating the benefits of CRF, but this potential reverse‐causation bias has not been routinely accounted for in earlier studies. Finally, smoking has both an immediate^23^ and long‐term^24^ detrimental impact on CRF and is also related to lower body weight. While restriction to never‐smokers as a measure to remove residual confounding is frequently applied in studies on BMI and mortality,^1^, ^3^ this is not a common practice for studies on CRF‐related mortality.

Determining whether higher CRF attenuates or eliminates the excess mortality risk associated with obesity has substantial implications for public health messaging and clinical counseling. In this study we aim to overcome the limitations in previous studies of the fat‐but‐fit hypothesis by implementing progressively more conservative approaches to address reverse‐causation bias, misclassification of body composition, and residual confounding from smoking.

## Methods

### Ethics Approval

All procedures performed were in accordance and with the 1964 Declaration of Helsinki and its later amendments or comparable ethical standards, and ethical approval was obtained from North West Multi‐centre Research Ethics Committee (REC reference: 11/NW/03820). Informed consent was obtained from all individual participants included in the study.

### Data Source and Study Population

We used data from the UK Biobank Resource (application number 29717). The UK Biobank is a population‐based observational cohort designed to improve the prevention, diagnosis, and treatment of chronic diseases. Between 2006 and 2010, a total of 502 682 participants (5.5% of the invited) aged 37 to 82 years were recruited via 22 assessment centers across England, Wales, and Scotland. From 2009, the baseline examination was extended to include a submaximal stationary bicycle test to estimate CRF at 5 test centers in England (n=78 968). In addition, a subset of the UK Biobank cohort was invited to repeat all measurements between 2012 and 2013, which included the bicycle test (n=20 209, 1 England center only), leading to a total of 99 177 bicycle test performances. Participants gave written informed consent before data collection, which included an electronic questionnaire, a wide variety of physical measurements, biological sampling, and permission to link with electronic registries. Ethical approval was obtained by the North West Research Ethics Committee. Full details of the protocol are available elsewhere.^25^ Data from the UK Biobank are available to researchers after registration at the UK Biobank server.^26^ The data cleaning and coding used to generate the findings of this study are available from the corresponding author on reasonable request.

### Cardiorespiratory Fitness

CRF was assessed using a stationary bicycle ergometer (eBike Comfort Ergometer, GE Healthcare). Based on individual risk classification,^27^ participants were assigned to a protocol consisting of graded cycling to 50% of predicted maximal workload, graded cycling to 30% of predicted maximal workload, cycling at constant workload, or at rest measurement only (we excluded the latter from this study). Maximal workload was predicted from age, sex, height, weight, and resting heart rate.^27^

Graded testing started with a 2‐minute constant workload of 30 W for women and 40 W for men, with the workload gradually increasing from minute 2 to 6 and ending with a 1‐minute rest. Graded testing was terminated prematurely if the participant wanted to stop, experienced discomfort, or their heart rate reached a predefined safety level. Graded exercise tests constituted 94% of the data. Time‐stamped measurements of heart rate and workload were used to derive individual watt–heart rate equations using the following order of priority: (1) 4‐minute graded workload; (2) 2‐minute constant workload if no graded data were recorded; or (3) 6‐minute constant workload. For individuals with constant workload data only, we used the mean heart rate of the final 3 data points coupled with resting heart rates obtained from measurements of blood pressure. From individual watt–heart rate equations we extrapolated the maximal workload (watt max) using the formulae 208−(0.7×age) to estimate maximal heart rate.^28^ Time‐stamped data were quality checked before calculation of slopes and constants and we excluded individuals with a negative or zero watt–heart rate slope. We derived 2 measures of CRF: normalized by body weight (W/kg) and by kilograms of FFM (W/kg FFM). We excluded individuals with W/kg FFM above the 99th percentile of the sample distribution because of a very long right‐tailed distribution. Repeated assessment of CRF was available in a subsample of 1851 individuals free from chronic diseases at both time points. The test‐retest Pearson correlations (median 2.9 years from baseline) were 0.72 for W/kg and 0.64 for W/kg FFM, with mean biases of 0.07 W/kg (95% confidence limits of agreement, −1.86 to 1.20) and 0.04 W/kg FFM (95% limits of agreemet, −1.30 to 1.40), respectively. The first assessment of CRF was used as the baseline in this study.

### Adiposity

Anthropometric measurements were taken by trained staff using standardized procedures. Height was measured using a stadiometer (Seca 202, Seca) and weight and body composition were measured using an electronic scale with bioimpedance (Tanita BC‐418MA Body Composition Analyser, Tanita). BMI was calculated as weight in kilograms divided by height in meters squared. Waist circumference was measured by a tape at the level of the umbilicus.

### Other Variables

Age was calculated as the difference between date of birth and date of baseline assessment. Ethnicity (White or others), education (no qualifications, no college/university degree, or college/university degree), living with partner (yes or no), and employment status (employed or unemployed) were self‐reported. The Townsend deprivation index was used as a marker of area‐level socioeconomic status, derived from postcode of residence at baseline and census data on housing, employment, social class, and car availability. Frequency of alcohol intake (never, previous, current and <3 times per week, current and ≥3 times per week), smoking status (current, former, never), television viewing, and diet were also self‐reported. We created a dichotomous variable summarizing dietary patterns based on meeting at least 2 of 3 healthy eating targets: (1) ≤3 weekly servings of red meat and ≤1 servings per week of processed meat^29^; (2) ≥2 servings per week of fish including at least 1 with oily fish^30^; and (3) ≥5 servings per day of fruits and vegetables.^29^ Baseline health status (cancer [other than nonmelanoma skin cancer], cardiovascular disease [CVD], asthma, history of depression, women taking hormone replacement therapy, statins medication, β‐blocker medication, calcium channel blocker medication, diabetes mellitus [nonspecific but excluding gestational diabetes mellitus], and hypertension) were extracted from a combination of self‐report, verbal interview with a trained nurse, and hospital records. Clinical measurements were used to flag individuals with unknown or unidentified hypertension (systolic blood pressure ≥140 mm Hg or diastolic blood pressure ≥90 mm Hg) or type 2 diabetes mellitus (glycated hemoglobin ≥48 mmol/L). Detailed information about variable extraction and coding is provided in Table S1.

### Statistical Analysis

Date of death was obtained from death certificates held by the National Health Service Information Centre for participants from England and Wales and the National Health Service Central Register Scotland for participants from Scotland. Patient‐years were calculated from the date attending the assessment center to the date of death, emigration, loss to follow‐up, or January 31, 2018, whichever came first. We excluded participants reporting the following conditions at baseline: prevalent chronic neurological degenerative problems, chronic widespread pain, chronic respiratory diseases (including chronic obstructive pulmonary disease), liver failure or cirrhosis, psychological or psychiatric problems, substance abuse or dependency, or eating disorders. We also excluded participants with a BMI <18.5 kg/m^2^, pregnant women, and individuals with missing height, weight, body fat percentage (BF%), or FFM, leading to 4208 exclusions in total (Figure S1).

CRF cutoffs were created from quintiles as the bottom 20% (unfit), 20% to ≤60% (medium fit), and >60% (fit) of the sex‐age (in 10‐year strata)^11^ sample distribution among individuals free from cancer and CVD and with >2 years of follow‐up (cutoffs presented in Table S2). These categories were combined with WHO BMI categories (normal weight [18.50–24.99], overweight [≥25], and obese [≥30]), yielding 9 fitness‐fatness combinations. In the absence of established cutoffs for BF% we modeled BF% categories on the sex‐specific distribution among individuals free from cancer and CVD (ie, 20% obese women would yield 20% in the high BF% category) and combined these with CRF.

Fitness‐BMI and fitness‐BF% categories were modeled for all‐cause mortality using Cox proportional hazard regression with age as the time scale and the normal‐weight/fit category as the reference. Hazard ratios (HRs) and 95% CIs were estimated from models adjusting for sex, Townsend index, education, partner status, ethnicity, employment status, diet pattern, alcohol intake, smoking status, television viewing, depression, asthma, hormone replacement therapy (women only), β‐blockers, calcium channel blockers, statins, hypertension, and diabetes mellitus. This model was repeated using gradually more conservative approaches to control for reverse causality, residual confounding from smoking, and misclassification of body composition. Model 1 is based on fitness in W/kg combined with BMI categories, adjusting for prevalent cancer and CVD. We consider this our base model because it is liberal (no restrictions) and it is a common analytical approach in earlier studies.^6^, ^11^, ^14^ Model 2 is based on model 1, but starting follow‐up 2 years after baseline and excluding individuals with cancer or CVD; model 3 is based on model 2 plus restricting to never‐smokers; model 4 is based on fitness in W/kg FFM combined with BF% categories, starting follow‐up 2 years after baseline and excluding individuals with cancer or CVD; and model 5 is based on model 4 plus restricting to never‐smokers.

Because women are underrepresented in the research on CRF,^2^, ^5^ we repeated our analyses stratified by sex. Cause‐specific associations with CVD mortality (*International Statistical Classification of Diseases, Tenth Revision* [*ICD‐10*] codes I05–I89.9) and cancer mortality (*ICD‐10* codes C‐D48) were determined using the subdistribution method by Fine and Gray^31^ with all other causes than the event of interest modeled as competing events. We also repeated our analyses with restriction to participants with a graded exercise test, stratified by age group (<60 and ≥60 years), using waist circumference to define adiposity, and after omitting control for the potential mediators (diabetes mellitus, hypertension, statins, β‐blockers, and calcium channel blockers). We did not proceed with stratified analysis among never‐smokers as the case count was considered insufficient with 9 exposure categories.

Missing covariates were imputed using chained equations with 20 data sets generated. The variable with the greatest proportion of missing data was dietary pattern (3.4% missing). The proportional hazards assumption was verified using log‐log plots and Schoenfeld residuals plotted against follow‐up time. Data were analyzed using Stata 16 (StataCorp LLC).

## Results

We included 77 169 participants (53% women) with a mean age of 57.8 years (8.2 years) at baseline (Table 1). During a median of 7.7 years (range, 0.2–8.2 years) of follow‐up, 1731 participants died (2.2%). Cause‐specific mortality was 73% cancer (488 cases) and 14% CVD (90 cases) among women and 59% cancer (633 cases) and 22% CVD (237 cases) among men. Unfit participants had more body fat and less FFM than fit participants within all categories of BMI (Table 2). In analysis of independent associations, higher fitness was associated with lower mortality in men and women when adjusting for CVD/cancer. When more conservative models were used this association was robust in men, but the dose‐response pattern was substantially changed in women (Table 3). Likelihood ratio tests supported sex‐by‐fitness multiplicative interactions in crude (*P*=0.05) but not in multivariable‐adjusted models (*P*>0.23). Conversely, BMI‐ and BF%‐categories associations with mortality increased in magnitude with more conservative models in women but not in men. Cross‐tabulation of BMI and BF% categories and fitness W/kg and W/FFM categories revealed that 73% of individuals were assigned the same adiposity category while 86% were assigned to the same fitness category (Tables S3 and S4).

**Table 1:** Baseline Characteristics of 77 169 Included Participants From UK Biobank

	Low CRF (≤20% of W/kg FFM)* n=15 581	Medium CRF (20% to ≤60% of W/kg FFM)* n=30 874	High CRF (>60% of W/kg FFM)* n=30 714	*P* Value
Age, y	58.4 (8.4)	57.9 (8.2)	57.5 (8.1)	<0.001
Women, %	52	53	53	0.52
CRF, W	108.5 (47.4)	168.9 (60.7)	243.9 (81.8)	<0.001
W/kg FFM	2.0 (0.6)	3.1 (0.6)	4.5 (0.8)	<0.001
W/kg body mass	1.4 (0.5)	2.2 (0.6)	3.1 (0.8)	<0.001
Body fat, %	30.9 (8.3)	31.2 (8.2)	31.1 (8.4)	0.001
Weight, kg	76.6 (16.3)	77.3 (15.4)	78.4 (14.6)	<0.001
BMI	27.1 (4.9)	27.1 (4.4)	27.1 (4.2)	0.37
Weight status, %				<0.001
Normal weight	37.7	34.7	33.8	
Overweight	38.7	43.5	45.4	
Obese	23.6	21.9	20.8	
Townsend deprivation index	−1.0 (3.1)	−1.3 (2.9)	−1.5 (2.8)	<0.001
Television viewing, h/d	2.9 (1.7)	2.7 (1.6)	2.5 (1.5)	<0.001
Education, %				<0.001
No qualifications	15.6	12.2	9.7	
Not College/university degree	51.2	51.1	47.5	
College/university degree	33.2	36.8	42.8	
White race, %	88.1	92.1	95.0	<0.001
Living with partner, %	69.9	73.3	74.4	<0.001
Employed, %	51.7	56.1	59.1	<0.001
Alcohol consumption, %				<0.001
Never	6.0	4.6	2.8	
Former	3.6	3.0	2.5	
Current, <3 times per wk	51.1	49.2	45.4	
Current, ≥3 times per wk	39.4	43.3	49.3	
Healthy dietary pattern (meeting at least 2 targets), %^†^	67.3	69.6	72.3	<0.001
Smoking status, %				<0.001
Never	59.7	57.9	55.7	
Former	31.8	34.4	36.3	
Current	8.6	7.7	8.1	
Hormone replacement treatment (%, women only)	3.4	3.5	3.8	0.054
β‐Blocker use, %	4.9	3.8	7.2	<0.001
Calcium channel blocker use, %	10.4	6.9	6.1	<0.001
Statins use, %	18.3	16.1	15.0	<0.001
Depression, %	4.8	4.9	5.5	<0.001
Asthma, %	10.9	11.1	10.3	0.01
Diabetes mellitus, %	7.3	5.4	4.2	<0.001
Hypertension, %	64.2	52.6	46.2	<0.001
CVD, %	4.7	3.7	3.8	<0.001
Cancer, %	9.6	9.7	9.2	0.10

Number varies from 74 557 (healthy diet pattern) to 77 169 because of missing data. BMI indicates body mass index; FFM, fat‐free mass; and W/kg, watts per kilogram.

* Cardiorespiratory fitness (CRF) cutoffs based on age (in 10‐year strata) and sex‐specific distribution in 66 943 participants free from cardiovascular disease (CVD) and cancer at baseline and with at least 2 years of observation time.

^†^ Healthy dietary pattern is based on meeting at least 2 of 3 healthy eating targets related to food types: (1) ≤3 weekly servings of red meat and ≤1 servings per week of processed meat; (2) ≥2 servings per week of fish including at least 1 with oily fish; and (3) ≥5 servings per day of fruits and vegetables.

**Table 2:** Baseline Characteristics by CRF‐Fatness Categories (Using W/kg Body Weight and BMI Categories)

	Age, y	Watt	CRF/kg	CRF/FFM	BMI	Waist Circumference	Weight, kg	BF%	FFM%
Normal weight/unfit	58.2 (8.5)	87.0 (35.0)	1.3 (0.4)	1.8 (0.5)	22.8 (1.6)	79.2 (8.4)	64.2 (8.4)	27.4 (7.1)	72.6 (7.1)
Normal weight‐medium fit	57.6 (8.4)	135.1 (46.9)	2.1 (0.5)	2.8 (0.6)	22.9 (1.5)	79.0 (8.3)	64.3 (8.4)	27.6 (6.8)	72.4 (6.8)
Normal weight‐fit	56.7 (8.2)	206.0 (70.2)	3.1 (0.8)	4.2 (0.9)	22.8 (1.5)	78.6 (8.1)	65.3 (8.5)	26.3 (7.0 )	73.7 (7.0)
*P value*	<0.001	<0.001	<0.001	<0.001	0.50	<0.001	<0.001	<0.001	<0.001

Overweight‐unfit	59.0 (8.3)	111.6 (45.7)	1.4 (0.5)	2.1 (0.6)	27.4 (1.4)	91.5 (8.7)	77.8 (9.6)	32.2 (7.2)	67.8 (7.2)
Overweight‐medium fit	58.3 (8.2)	176.3 (58.7)	2.2 (0.6)	3.2 (0.6)	27.3 (1.4)	91.4 (8.4)	78.6 (9.7)	31.5 (7.2)	68.5 (7.2)
Overweight‐fit	58.0 (8.1)	256.8 (79.7)	3.2 (0.8)	4.5 (0.8)	27.1 (1.4)	90.9 (8.1)	79.5 (9.4)	30.1 (7.3)	69.9 (7.3)
*P value*	<0.001	<0.001	<0.001	<0.001	<0.001	<0.001	<0.001	<0.001	<0.001

Obese‐unfit	57.8 (8.2)	136.8 (58.4)	1.4 (0.5)	2.2 (0.7)	34.4 (4.0)	106.3 (11.3)	97.1 (14.6)	38.7 (7.7)	61.3 (7.7)
Obese‐medium fit	57.7 (8.1)	207.9 (69.0)	2.1 (0.5)	3.4 (0.6)	33.4 (3.2)	104.4 (10.2)	95.5 (13.0)	37.9 (7.7)	62.1 (7.7)
Obese‐fit	58.1 (7.7)	288.9 (91.0)	3.0 (0.7)	4.7 (0.8)	32.7 (2.6)	102.8 (9.5)	94.5 (12.1)	36.6 (7.7)	63.4 (7.7)
*P* value	0.02	<0.001	<0.001	<0.001	<0.001	<0.001	<0.001	<0.001	<0.001

N=77 169. Values are mean (SD). BF% indicates % body fat; BMI, body mass index; CRF, cardiorespiratory fitness; FFM, fat‐free mass; and W/kg, watts per kilogram. *P*‐values are based on an analysis of variance within categories of BMI.

**Table 3:** "Independent" Associations Between CRF, BMI, and BF% With All‐Cause Mortality With Progressively More Conservative Models

	Full Sample				Free From CVD/Cancer at Baseline and Removing Early Follow‐Up				Never‐Smokers, Free From CVD/Cancer at Baseline and Removing Early Follow‐Up			
	Women (n=40 737, 665 Deaths)		Men (n=36 432, 1066 Deaths)		Women (n=35 080, 385 Deaths)		Men (n=31 863, 621 Deaths)		Women (n=22 036, 186 Deaths)		Men (n=16 848, 237 Deaths)	
	Multivariable‐Adjusted Including BF%				Multivariable‐Adjusted Including BF%				Multivariable‐Adjusted Including BF%			
W/kg body weight												
Least fit quintile	Reference		Reference		Reference		Reference		Reference		Reference	
2	0.85 (0.68–1.07)		0.82 (0.69–0.97)		0.86 (0.63–1.16)		0.77 (0.61–0.96)		0.95 (0.61–1.48)		0.66 (0.45–0.97)	
3	0.86 (0.68–1.08)		0.75 (0.62–0.89)		0.84 (0.61–1.15)		0.71 (0.56–0.90)		0.98 (0.62–1.56)		0.77 (0.53–1.12)	
4	0.71 (0.55–0.91)		0.69 (0.57–0.83)		0.75 (0.54–1.05)		0.66 (0.52–0.85)		1.08 (0.67–1.72)		0.70 (0.47–1.04)	
Most fit quintile	0.83 (0.65–1.06)		0.65 (0.53–0.80)		1.14 (0.83–1.56)		0.60 (0.46–0.79)		1.44 (0.91–2.27)		0.56 (0.36–0.86)	
W/kg FFM												
Least fit quintile	Reference		Reference		Reference		Reference		Reference		Reference	
2	0.90 (0.72–1.13)		0.82 (0.69–0.97)		0.98 (0.72–1.33)		0.73 (0.59–0.92)		0.97 (0.61–1.53)		0.71 (0.49–1.04)	
3	0.76 (0.60–0.97)		0.78 (0.65–0.94)		0.74 (0.53–1.03)		0.77 (0.61–0.97)		1.04 (0.66–1.64)		0.75 (0.51–1.09)	
4	0.85 (0.67–1.08)		0.68 (0.56–0.82)		0.97 (0.71–1.33)		0.63 (0.49–0.82)		1.15 (0.73–1.82)		0.79 (0.53–1.16)	
Most fit quintile	0.79 (0.62–1.01)		0.67 (0.55–0.82)		1.02 (0.75–1.40)		0.63 (0.48–0.81)		1.29 (0.82–2.04)		0.65 (0.42–0.99)	

	Multivariable‐Adjusted Including CRF/FFM				Multivariable‐Adjusted Including CRF/FFM				Multivariable‐Adjusted Including CRF/FFM			
BMI status												
Normal weight	Reference		Reference		Reference		Reference		Reference		Reference	
Overweight	1.02 (0.85–1.22)		0.99 (0.85–1.16)		0.99 (0.78–1.26)		1.02 (0.83–1.25)		1.00 (0.71, 1.41)		1.25 (0.91, 1.72)	
Obese	1.19 (0.96–1.47)		1.11 (0.93–1.33)		1.23 (0.93–1.62)		1.10 (0.86–1.39)		1.30 (0.88, 1.93)		1.14 (0.77, 1.70)	
BF% status*												
Low BF					Reference		Reference		Reference		Reference	
Medium BF					0.99 (0.78–1.25)		0.97 (0.78–1.19)		0.91 (0.64, 1.30)		1.04 (0.75, 1.43)	
High BF					1.14 (0.86–1.50)		1.01 (0.80–1.29)		1.32 (0.90, 1.94)		0.89 (0.60, 1.33)	

Hazard ratios with 95% CI. Multivariable‐adjusted: age (time scale), sex, Townsend index, education, partner status, ethnicity, employment status, diet pattern, alcohol intake, smoking status, television viewing, depression, asthma, hormone replacement therapy (women only), β‐blockers, calcium channel blockers, statins, hypertension, and diabetes mellitus (analysis in full sample additionally adjusted for prevalent cardiovascular disease [CVD] and cancer). BMI indicates body mass index; CRF, cardiorespiratory fitness; FFM, fat‐free mass; and W/kg, watts per kilogram.

* Body fat percentage (BF%) status was defined based on distribution among CVD and cancer‐free participants at baseline, so was not analyzed in the full sample.

### Combined CRF‐Fatness Categories and Mortality

In our base model (normalizing by body weight and adjusting for CVD/cancer), all fitness‐fatness combinations were associated with an increased risk of mortality as compared with the normal weight–fit reference category. Compared with the reference, HRs were 1.66 (95% CI, 1.30–2.10), 1.55 (95% CI, 1.19–1.92), and 1.76 (95% CI, 1.41–2.20) for normal weight‐unfit, overweight‐unfit, and obese‐unfit categories, respectively (Table 4). These associations were substantially attenuated among the overweight‐fit (1.23 [95% CI, 1.01–1.49]) and the obese‐fit (1.27 [95% CI, 0.98–1.63]). This pattern of higher risk in the unfit, irrespective of the level of adiposity, was consistent for all models when normalizing fitness by total body weight and categorizing fatness using BMI (models 1–3) although with some attenuation of HRs in more conservative models. For example, when restricted to never‐smokers (model 3), HRs were 1.41 (95% CI, 0.89–2.21), 1.42 (95% CI, 0.94–2.15), and 1.42 (95% CI, 0.91–2.22) among normal weight‐unfit, overweight‐unfit and obese‐unfit, respectively. The pattern of fitness moderating the association between adiposity and mortality was partly changed when CRF was normalized by FFM and combined with BF% (models 4 and 5). Compared with the reference, the risk of mortality was now lower among the overweight‐fit (model 4: HR, 0.77 [95% CI, 0.53–1.11]) and obese‐fit (model 4: HR, 0.76 [95% CI, 0.56–1.03]), albeit with CIs included unity.

**Table 4:** Associations Between CRF‐Fatness Combinations and All‐Cause Mortality

	Model 1 CRF/kg+BMI All Participants (Adjusting for Prevalent Cancer/CVD)	Model 2 CRF/kg+BMI Free From CVD/Cancer at Baseline and Removing Early Follow‐Up	Model 3 CRF/kg+BMI Never‐Smokers, Free From CVD/Cancer at Baseline and Removing Early Follow‐Up	Model 4 CRF/FFM+BF% Free From CVD/Cancer at Baseline and Removing Early Follow‐Up	Model 5 CRF/FFM+BF% Never‐Smokers, Free From CVD/Cancer at Baseline and Removing Early Follow‐Up
n (deaths)	77 169 (1731)	66 943 (1006)	38 884 (423)	66 943 (1006)	38 884 (423)
Normal weight‐unfit	1.66 (1.30–2.10)	1.38 (1.01–1.89)	1.41 (0.89–2.21)	1.01 (0.75–1.37)	0.93 (0.60–1.44)
n (deaths)	4373 (117)	3795 (63)	2411 (31)	4975 (71)	3192 (34)
Normal weight‐medium fit	1.46 (1.19–1.80)	1.26 (0.96–1.64)	1.03 (0.69–1.55)	0.85 (0.64–1.11)	0.68 (0.45–1.02)
n (deaths)	9585 (205)	8340 (113)	5271 (44)	9267 (99)	5763 (40)
Normal weight‐fit	Ref	Ref	Ref	Ref	Ref
n (deaths)	13 003 (167)	11 516 (109)	7017 (50)	9551 (107)	5744 (53)
Overweight‐unfit	1.55 (1.19–1.92)	1.44 (1.10–1.90)	1.42 (0.94–2.15)	1.15 (0.87–1.51)	1.01 (0.68–1.52)
n (deaths)	6124 (190)	5236 (112)	3102 (48)	5359 (115)	3164 (49)
Overweight‐medium fit	1.21 (0.99–1.46)	1.05 (0.82–1.34)	1.15 (0.80–1.66)	0.86 (0.67–1.10)	0.84 (0.58–1.20)
n (deaths)	13 887 (300)	12 058 (167)	6886 (76)	11 654 (167)	6749 (76)
Overweight‐fit	1.23 (1.01–1.49)	1.16 (0.91–1.48)	1.24 (0.86–1.78)	0.86 (0.67–1.10)	0.77 (0.53–1.11)
n (deaths)	13 384 (276)	11 708 (170)	6382 (70)	12 005 (163)	6614 (63)
Obese‐unfit	1.76 (1.41–2.20)	1.65 (1.25–2.20)	1.42 (0.91–2.22)	1.33 (0.99–1.79)	0.95 (0.59–1.54)
n (deaths)	5164 (184)	4354 (107)	2536 (38)	3051 (88)	1742 (28)
Obese‐medium fit	1.40 (1.13–1.73	1.25 (0.95–1.65)	1.21 (0.79–1.86)	1.06 (0.81–1.39)	0.91 (0.60–1.39)
n (deaths)	7426 (190)	6383 (109)	3443 (40)	5860 (121)	3188 (44)
Obese‐fit	1.27 (0.98–1.63	1.10 (0.79–1.53)	1.48 (0.91–2.40)	0.76 (0.56–1.03)	0.94 (0.61–1.46)
n (deaths)	4223 (102)	3553 (56)	1836 (26)	5221 (75)	2728 (36)

Hazard ratios with 95% CIs. Adjusted for age (time scale), sex, Townsend index, education, partner status, ethnicity, employment status, diet pattern, alcohol intake, smoking status, television viewing, depression, asthma, hormone replacement therapy (women only), β‐blockers, calcium channel blockers, statins, hypertension, and diabetes mellitus (including cardiovascular disease [CVD] and cancer in model 1 and 2). Follow‐up was commenced 2 years after baseline in model 2–5. BF% indicates body fat percentage; BMI, body mass index; CRF, cardiorespiratory fitness in watts; and FFM, fat‐free mass.

### Fitness‐Fatness Associations With Mortality Stratified by Sex

In men, restricting the analysis to individuals free from CVD/cancer at baseline and removing the first 2 years of follow‐up (model 2) did not materially change the results compared with the base model (Figure, Table S5). However, associations were attenuated for most categories when further accounting for misclassification of body composition (model 4) with HRs of 1.78 (95% CI, 1.17–2.71) and 0.94 (95% CI, 0.60–1.48) for the obese‐unfit and obese‐fit, groups respectively. In contrast, the effect of using more conservative models was much more pronounced in women. Compared with the base model, which suggested fitness moderated the association between BMI and mortality, this pattern was less clear when accounting for reverse‐causation bias in model 2 (Figure, Table S5). When further accounting for body composition misclassification, the pattern was completely changed, with no elevated risk in unfit‐obese women (HR, 1.09 [95% CI, 0.68–1.77]), compared with the reference. In both men and women, being unfit‐normal weight was associated with an increased mortality risk in the base model (Figure). In men, the association was attenuated by ≈30% with more conservative models (model 4: HR, 1.71 [95% CI, 1.10–2.67]), whereas the association was reversed in women (model 4: HR, 0.63 [95% CI, 0.61–0.98]). The importance of high fitness is illustrated by obese‐fit men having half the risk of premature mortality compared with the normal weight‐unfit phenotype (model 4: HR, 0.55 [95% CI, 0.36–0.85]). The risk was similar in obese‐fit and normal weight‐unfit women (model 4: HR, 1.08 [95% CI, 0.65–1.79]).

Restricting analysis to individuals with a graded exercise test (Table S6), stratifying the sample at 60 years of age (Table S7), using waist circumference as a marker of central adiposity (Table S8), or omitting control from potential mediating variables (Table S9) did not change the overall pattern of associations for CRF‐fatness combinations. Analysis of CVD and cancer‐specific mortality revealed stronger associations for CVD than for cancer mortality (Table S10).

## Discussion

In analysis of 77 169 middle‐aged men and women we observed that CRF modified the association between obesity and mortality such that, compared with normal weight individuals with high CRF, the risk of mortality was only higher in those who were obese and unfit but not among those who were obese and fit. Further, obese and fit men had lower mortality than normal weight‐unfit men. Importantly, the magnitude and association‐pattern between CRF‐fatness combinations and mortality observed in our base model was not robust to analytical approaches accounting for reverse‐causation bias and misclassification of body composition, particularly among women. Assuming the impact of using more conservative models observed in our study is generalizable to other cohorts and populations, this suggests that results from earlier studies on the fat‐but‐fit hypothesis may have been inflated as their analytical approaches closely resemble our base model.^6^, ^7^, ^10^, ^11^, ^12^, ^13^, ^14^, ^15^, ^32^ Appropriate handling of these biases is needed to determine cost‐benefit ratios among competing public health strategies.

### Comparison With Previous Research

Previous studies have shown a wide range of estimates for the association between CRF‐fatness combinations and mortality. Compared with the normal weight‐fit reference, the risk (HR) among normal weight‐unfit were 2.0 in VETS (Veterans Exercise Testing Study)^15^ and 1.5 and 2.2 in 2 nonoverlapping samples from the ACLS.^7^, ^13^ In these studies, the risk among the obese‐unfit ranged from 1.6 to 3.1 and from 0.5 to 1.1 among the obese‐fit.^7^, ^13^, ^15^ Using finer‐grained categories of BMI in a larger analysis from VETS highlighted the importance of CRF across the full spectrum of BMI.^33^ These differences in magnitude of association are likely explained by different samples, measurement methods, and analytical approaches, but 2 consistent observations can be made: first, the importance of CRF irrespective of weight status; and second, in those with high CRF, obesity did not increase the risk of mortality. The results of our base model were within the range of findings from these earlier studies but were attenuated when more conservative models were used, particularly among women.

We observed that excluding individuals with prevalent CVD, those with cancer, and starting follow‐up 2 years after baseline attenuated HRs slightly in all CRF‐fatness categories, but with a more pronounced attenuation in the normal weight‐unfit category. This may be explained by residual confounding from disease status not accounted for through the statistical adjustment. Accounting for these mechanisms may be particularly prudent among those with low body weight as their disease status may be more progressed. The notion that statistical adjustment for prevalent disease may be insufficient to fully handle reverse‐causation bias has been suggested elsewhere.^34^, ^35^ In analysis stratified by sex, attenuated HRs among the normal weight‐unfit appeared driven by changes in estimates among women, while estimates were virtually unaffected by this restriction in men. It is unclear why women should be more susceptible to reverse causation bias than men. In an analysis from the ACLS, removal of deaths within the first 5 years after baseline did not materially change estimates or the association‐pattern, but very few deaths in each exposure combination calls for a cautious interpretation.^8^ We accounted for misclassification of body composition by using a direct measure of body fat rather than BMI and by normalizing CRF by FFM to avoid using a measure of fitness that is confounded by total body mass.^20^ We suspect the attenuated HRs among normal weight‐unfit men when using body fat instead of BMI may be a result of avoiding misclassification of individuals into low BMI because of low FFM, which would likely bias estimates away from the null as low FFM is associated with a higher mortality risk,^36^ possibly caused by underlying subclinical disease.^21^, ^37^, ^38^ In contrast, a BMI >30 kg/m^2^ is unlikely to purely reflect high FFM in middle‐aged individuals.^36^ We therefore suggest that the attenuation among obese‐unfit men reflects reclassification of some individuals with high total body weight, but appropriate FFM, into other fitness categories because of normalization to FFM rather than total body weight. Accounting for body composition misclassification had a larger impact on women than men, with analysis based on FFM and BF% completely altering the dose‐response pattern for CRF. It is possible that differences in body fat distribution between men and women may influence the modifying role of CRF. However, using waist circumference as the adiposity metric did not change the pattern of associations. In a cohort of women from the BALL ST (Ball State Adult Fitness Longitudinal Lifestyle Study), CRF normalized by FFM, but not total body weight, was associated with lower mortality.^39^ We suspected that restriction to never‐smokers would influence estimates of mortality risk as smoking is related to increased risk of mortality but decreased body weight and lower fitness.^3^, ^24^ However, this was not the case in analysis already accounting for prevalent CVD/cancer and early mortality. This may be a result of the low prevalence of current smokers in the cohort. Unfortunately, we were unable to examine the impact of this restriction in sex‐stratified analysis because of insufficient information in all exposure categories. Examination of the impact of smoking‐related residual confounding warrants further attention in CRF research in general.

Contrary to our expectations, we did not observe a robust inverse dose‐response relationship between CRF and mortality in women. Clinical studies suggest that exercise leading to greater CRF is associated with improved metabolic regulation in both women and men,^40^ but the majority of studies linking CRF with mortality are conducted in men.^2^ Available studies restricted to women have suggested dose‐response associations with a single measurement of CRF performed at baseline^7^, ^41^ and lower mortality in women who increased their fitness over time.^42^ Whether CRF modifies the obesity‐mortality association is particularly under‐researched in women. Previous studies are limited by small samples or analytical approaches resembling that of our base model.^7^, ^11^, ^14^, ^32^ Based on available observational and experimental evidence, we find it unlikely that higher CRF should not yield CVD and mortality benefits in women. However, a recent large twin study including 4260 male twin pairs observed no difference in risk of CVD or mortality between twins that were discordant in their fitness,^43^ suggesting that confounding may explain the association observed in conventional observational analysis. This is an important study because the twin design should remove genetic confounding and may also reduce confounding from social factors. We therefore encourage researchers to apply innovative study designs to confirm the protective role of CRF on mortality in both men and women.

### Implications

To further advance our understanding of how CRF may offset the increased risk of premature mortality with obesity, and the dose‐response pattern for CRF in general, we suggest future studies implement rigorous methodological and analytical strategies, which, based on our findings, should include normalization of fitness to FFM, using a direct measure of body fat; exclusion of individuals with prevalent disease; and implementing analysis left truncation to exclude early follow‐up time. We also suggest further studies perform sex‐stratified analyses and examine the robustness of their results in never‐smokers if case counts permit. The importance of CRF irrespective of the level of adiposity underscores the importance of: (1) monitoring individual and population CRF levels; (2) facilitating individual and population strategies for improving and maintaining good CRF; and (3) encourage those with high body weight to increase their physical activity or engage in aerobic exercise to improve CRF, which have health benefits independent of weight status.

### Limitations

We highlight the following limitations. First, the number of deaths was relatively modest, which prevented sex‐stratified results restricted to never‐smokers and other subgroups. Second, BF% and FFM were estimated from bioimpedance measurements, which, although with a strong correlation, comes with nontrivial individual error as compared with dual‐energy X‐ray absorptiometry.^44^ Bioimpedance measurement of body fat is influenced by height, cross‐sectional area, and ionic composition of the body.^45^ Further, as total body weight is equal to body fat plus FFM, the errors in measurements of BF% and FFM are not independent. Thereby, measurement error could result in misclassification of both the fatness and the fitness (when normalized to lean mass) component. Third, in contrast with the majority of earlier studies on the fat‐but‐fit hypothesis,^5^ we used a submaximal test to determine CRF. Submaximal fitness assessments are highly correlated with measured maximal oxygen uptake,^46^ but the validity of submaximal tests depends, among other factors, on the range of intensities at which data are collected. The maximal target intensity in the UK Biobank protocol was 50% of estimated watt‐max, which is lower than applied elsewhere.^47^, ^48^ We created individual work‐heart rate slopes and extrapolated these to estimated maximal heart rate, which also adds error. Error from submaximal fitness assessment may be exacerbated in current smokers.^23^ The fitness protocol in the UK Biobank was individualized based on clinical characteristics^27^ and it is unclear how these adaptations may impact the validity of the test. We are unaware of any formal validation of the UK Biobank fitness protocols but the well‐described strong relationship between ergometer workload, heart rate, and fitness and the clear dose‐response association in men provides face validity. Fourth, limiting reverse‐causation bias through the exclusion of individuals with prevalent CVD or cancer and removing early follow‐up time assumes this approach will remove bias, yet some have questioned the validity of this procedure.^49^ More work is needed to characterize optimal analytical strategies of mortality in cohort studies under different scenarios of age, disease prevalence, and duration of follow‐up. We excluded the first 2 years of follow‐up to limit the influence of reverse causation bias, but it is unclear whether this time frame is sufficient to fully remove bias.^1^, ^35^ Fifth, while we controlled for many demographic, behavioural, and clinically measured factors, the study is observational and we cannot exclude the risk of residual or unmeasured confounding and other biases. The UK Biobank is highly selected towards healthier individuals living in urban areas, which may affect the generalizability of exposure‐outcome associations.^50^, ^51^ If selection mechanisms leading to participation in the UK Biobank are not identical between men and women, this could be a potential explanation for the different association‐patterns observed.^52^

## Conclusions

Low CRF remained associated with an almost 2‐fold higher risk of premature mortality in men, irrespective of the level of adiposity, after accounting for several previously insufficiently addressed sources of bias. Obese‐fit men were not at an elevated risk of premature mortality compared with normal weight‐fit men and had a lower mortality than normal weight‐unfit men. In women, this pattern was evident in our base model but not in more conservative models, suggesting a need for further examinations of a potentially modifying role of CRF in the obesity‐mortality association among women. Authorities and clinicians should promote physical activity of sufficient intensity and frequency to improve CRF in individuals with low fitness irrespective of their weight status.

## Sources of Funding

The UK Biobank was supported by the Wellcome Trust, Medical Research Council, Department of Health, Scottish Government, and Northwest Regional Development Agency. It has also had funding from the Welsh Assembly Government and British Heart Foundation. The research was designed, conducted, analyzed, and interpreted by the authors entirely independently of the funding sources. Tarp was funded by the Research Council of Norway (grant 249932/F20), Grøntved by the European Research Council (starting grant 716657), Sanchez‐Lastra by the Xunta de Galicia (grant ED481A‐2017/213), and Ding by a Heart Foundation Australia Future Leader Fellowship (grant 101234) while contributing to this work. No funding directly supported the work.

## Disclosures

None.

**Figure 1:**
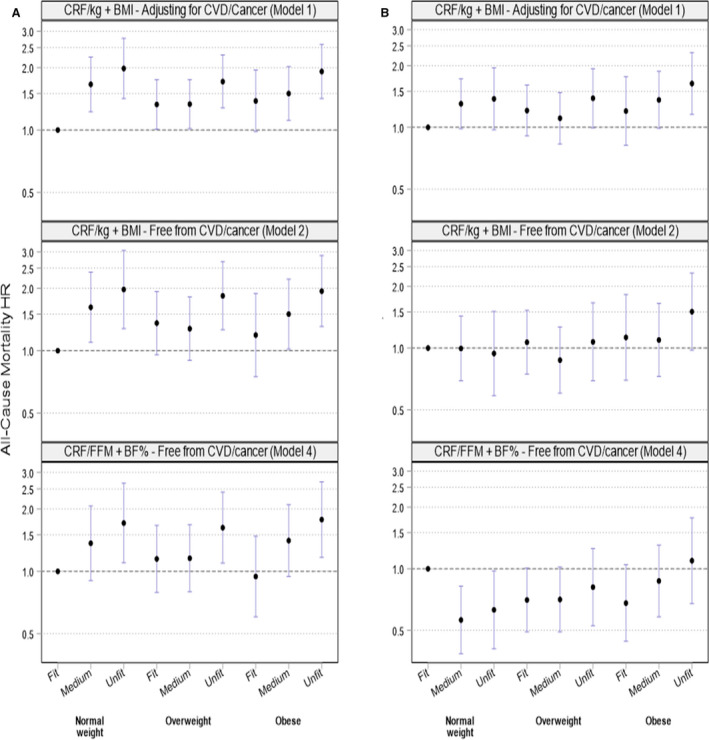
Sex‐stratified associations between cardiorespiratory fitness (CRF)‐fatness combinations and all‐cause mortality. Hazard ratios (HRs) with 95% CI. **A**, men, (**B**) women. Model 1: CRF in watts per kilograms (w/kg) and body mass index (BMI) categories. Including all participants and with adjustment for prevalent cardiovascular disease (CVD)/cancer (men: n=36 432, 1066 deaths; women: n=40 737, 665 deaths). Model 2: CRF in w/kg and BMI categories and participants free from prevalent CVD and cancer at baseline and with follow‐up commenced 2 years after the baseline examination (men: n=31 863, 621 deaths; women: n=35 080, 385 deaths). Model 4: CRF in watts per fat‐free mass (FFM) and body fat percentage (BF%) categories and participants free from prevalent CVD and cancer at baseline and with follow‐up commenced 2 years after the baseline examination (men: n=31 863, 621 deaths; women: n=35 080, 385 deaths). Exact HRs and CIs shown in Table S5. All models are adjusted for age (time scale), Townsend index, education, partner status, ethnicity, employment status, diet pattern, alcohol intake, smoking status, television viewing, depression, asthma, hormone replacement therapy (women only), β‐blockers, calcium channel blockers, statins, hypertension, and diabetes mellitus (model 1 additionally adjusting for prevalent CVD and cancer).

## Supporting information


Tables S1–S10
Figure S1
