# Correction to: Fitness, Fatness, and Mortality in Men and Women From the UK Biobank: Prospective Cohort Study

**DOI:** 10.1161/JAHA.121.020837

**Published:** 2021-04-06

In the article by Tarp et al, “Fitness, Fatness, and Mortality in Men and Women From the UK Biobank: Prospective Cohort Study,” which published on March 13, 2021 and appeared in the March 16, 2021 issue of the Journal (*J Am Heart Assoc*. 2021;10:e019605. DOI: 10.1161/JAHA.120.019605), corrections were needed.

The columns in the bottom half of Table 3 were misaligned, and have now been corrected. In Table 4, in the stub column, the line headings reading “Deaths, n” have been corrected to read “N (deaths)”. No change has been made to the data in those rows.

The publisher regrets the errors.

The footnotes have been added to the current online version of the article, which is available here: https://www.ahajournals.org/doi/10.1161/JAHA.120.019605.

