# Supplementary material for: Fitness, Fatness, and Mortality in Men and Women From the UK Biobank: Prospective Cohort Study
Source: J Am Heart Assoc. 2021 Mar 13;10(6):e019605. doi: 10.1161/JAHA.120.019605 (PMC8174221; doi:10.1161/JAHA.120.019605)
Supplement: Supplementary file 1 — Tables S1–S10 Figure S1 [file JAH3-10-e019605-s001.pdf]

SUPPLEMENTAL MATERIAL

Table S1. Description of variables included from UK Biobank Data Showcase

Item	UK Biobank Field ID(s)	Measurement item(s)	Definition/data coding (not listed if no changes made to original coding)	Notes
Date of Death	40000	(Death register) date of death		
Date lost to follow-up	191	(multiple sources) date which a person is believed to be lost to follow-up		http://biobank.ndph.ox.ac.uk/showcase/field.cgi?id=191
Primary cause of death	40001	(Death register) underlying/primary cause of death		http://biobank.ndph.ox.ac.uk/showcase/field.cgi?id=40001
Birthday	34 & 52	(Registry, updated by participant) year and month of birth	Date set to 15th for all participants	
Country	54	(Automatically acquired at participant consent) UK Biobank assessment centre		
Sex	31	(Registry, updated by participant)		
Date of attending UK Biobank assessment centre	53	(Acquired at UK Biobank Reception)	Wales = if `x'== 11003 | `x'==11022 | `x'==11023<br>Scotland = if `x'== 11005 | `x'==11004<br>England = if none of the above satisfied	http://biobank.ndph.ox.ac.uk/showcase/field.cgi?id=54
Tv-viewing	1070	(Questionnaire) self-reported time-use of tv-viewing	TV-viewing = 0.5 if `x'== 'less than 1h'	
Resting HR	102	Measured pulse rate during blood pressure readings		http://biobank.ndph.ox.ac.uk/showcase/showcase/docs/Bloodpressure.pdf
ECG/bike method for fitness test	6019	Bike method		http://biobank.ndph.ox.ac.uk/showcase/showcase/docs/Cardio.pdf
Completion status of test	6020	Completion status of test		http://biobank.ndph.ox.ac.uk/showcase/showcase/docs/Cardio.pdf
Cycling program category	6024	Cycling program based on individual risk		http://biobank.ndph.ox.ac.uk/showcase/showcase/docs/Cardio.pdf
Maximum workload during fitness test	6032	Maximum workload attained during the fitness test		http://biobank.ndph.ox.ac.uk/showcase/showcase/docs/Cardio.pdf
Maximum heart rate during fitness test	6033	Maximum heart rate recorded during bike exercise test		http://biobank.ndph.ox.ac.uk/showcase/showcase/docs/Cardio.pdf
Number of trend entries	6038	Measurements during exercise test		http://biobank.ndph.ox.ac.uk/showcase/showcase/docs/Cardio.pdf
Duration of fitness test	6039	Length of the exercise test		http://biobank.ndph.ox.ac.uk/showcase/showcase/docs/Cardio.pdf
Heart rate (from ECG)	5983	Heart rate from ECG		http://biobank.ndph.ox.ac.uk/showcase/showcase/docs/Cardio.pdf
Load (watt)	5984	Workload in watts		http://biobank.ndph.ox.ac.uk/showcase/showcase/docs/Cardio.pdf

Time	5986	Time during specific test-phase		http://biobank.ndph.ox.ac.uk/showcase/showcase/docs/Cardio.pdf
Phase name	5987	Name of test-phase		http://biobank.ndph.ox.ac.uk/showcase/showcase/docs/Cardio.pdf
Stage name	5988	Name of test-stage		http://biobank.ndph.ox.ac.uk/showcase/showcase/docs/Cardio.pdf
Waist circumference	48	Measured waist circumference		
Body mass index	21001	Measured height and weight		
Body fat %	23099	Body composition estimation by impedance measurement		
Oily Fish intake (servings/week)	1329	(Questionnaire) self-reported frequency of oily fish intake	Never = 0<br>less than once a week = 0.5<br>Once a week = 1<br>2-4 times a week = 3<br>5-6 times a week = 5.5<br>Once or more daily = 7	Included as part of diet pattern variable
Non-oily Fish intake (servings/week)	1339	(Questionnaire) self-reported frequency of non-oily fish intake	Never = 0<br>less than once a week = 0.5<br>Once a week = 1<br>2-4 times a week = 3<br>5-6 times a week = 5.5<br>Once or more daily = 7	Included as part of diet pattern variable
Fruit/vegetable intake (servings/week)	1289, 1299, 1309, 1319	(Questionnaire) self-reported frequency of cooked vegetable intake, salad/raw vegetable intake, fresh fruit intake, dried fruit intake	Less than one = 0.5<br>Otherwise, number of reported tablespoons (vegetables) and pieces (fruit) as reported	Included as part of diet pattern variable
Processed meat (servings/week)	1349	(Questionnaire) self-reported frequency of processed meat intake	Never = 0<br>less than once a week = 0.5<br>Once a week = 1<br>2-4 times a week = 3<br>5-6 times a week = 5.5<br>Once or more daily = 7	Included as part of diet pattern variable
Red meat (servings/week)	1369, 1379, 1389	(Questionnaire) self-reported frequency of beef, lamb/mutton and pork intake	Never = 0<br>less than once a week = 0.5<br>Once a week = 1<br>2-4 times a week = 3<br>5-6 times a week = 5.5<br>Once or more daily = 7	Included as part of diet pattern variable
Healthy diet pattern	Generated for this dataset	Composite of self-reported fish, fruit/vegetable and processed/red meat intake	1 if meeting 2 of the 3 food item targets<br>0 if not meeting 2 of the targets	Fish: >=2 servings/week, including 1 with oily fish<br>https://www.nhs.uk/live-well/eat-well/eight-tips-for-healthy-eating/<br><br>Fruit/vegetables: >=5 servings/day<br>https://www.nhs.uk/live-well/eat-well/5-a-day-what-counts/<br>+ doi: 10.1016/S2468-2667(18)30200-7

<table>
<tr><td></td><td></td><td></td><td></td><td>Processed/red meat intake: <= 3 servings of
red meat/week & <=1 serving of processed meat/week
https://www.ncbi.nlm.nih.gov/pubmed/30467019 +
doi: 10.1016/S2468-2667(18)30200-7</td></tr>
<tr><td>Education</td><td>6138</td><td>(Questionnaire) self-reported qualifications</td><td>No qualifications = if `x'== -7<br>A/AS/O/GCSE/CSE/NVQ/HND/HNC/Other professional qualifications = if `x'== 2 | `x'== 3 | `x'== 4 | `x'== 5 | `x'== 6
College/University degree = if `x'== 1</td><td>Original categories do not have a clear ordinal structure which accounts for all 7 answering options</td></tr>
<tr><td>Townsend</td><td>189</td><td>Townsend deprivation index calculated immediately prior to participant joining UK Biobank. Based on the preceding national census output areas. Each participant is assigned a score corresponding to the output area in which their postcode is located.</td><td></td><td></td></tr>
<tr><td>Partner status</td><td>Combinations of 709 and 6414</td><td>(Questionnaire) self-reported individuals (including yourself) living in household and relation to those individuals</td><td>Not married/living with partner = if n_709_0_0 == 1
Married/living with partner = if n_709_0_0 >1 & n_6414_0_0 - n_6414_0_4 == 1</td><td></td></tr>
<tr><td>Ethnicity</td><td>21000</td><td>(Questionnaire) amalgam of sequential branching questions</td><td>White = if `x'==1 | `x'==1001 | `x'==1002 | `x'==1003
Other = if above not satisfied</td><td></td></tr>
<tr><td>Employment</td><td>6142</td><td>(Questionnaire) self-reported 'current situation'</td><td>Employed = if `x'== 1</td><td></td></tr>
<tr><td>Smoking</td><td>20116</td><td>(Questionnaire) self-reported smoking-status</td><td></td><td></td></tr>
<tr><td>Alcohol</td><td>Combinations of 20117 & 1558</td><td>(Questionnaire) self-reported drinking status and drinking frequency</td><td>Never = n_20117_0_0 == 0
Previous = n_20117_0_0 == 1
Current, <3times/week = 2 if n_20117_0_0 == 2 & (n_1558_0_0 == 3 | n_1558_0_0 == 4 | n_1558_0_0 == 5
Current, ≥3times/week = 3 if n_20117_0_0 == 2 & (n_1558_0_0 == 1 | n_1558_0_0 ==2</td><td></td></tr>
<tr><td>Beta-blocker use</td><td>20003</td><td>(Interview) self-reported use of beta-blockers</td><td>Any of 235 codes listed in Verweij et al., 2017</td><td>doi: 10.1038/s41598-017-03062-8</td></tr>
<tr><td>Calcium-Channel inhibitor use</td><td>20003</td><td>(Interview) self-reported use of Calcium-Channel inhibitors</td><td>Any of 80 codes listed in Verweij et al., 2017</td><td>doi: 10.1038/s41598-017-03062-8</td></tr>
<tr><td>Statins use</td><td>20003</td><td>(Interview) self-reported use of statins</td><td>Atorvastatin, Fluvastatin, Pravastatin, Rosuvastatin, Simvastatin</td><td>https://www.nhs.uk/conditions/statins/</td></tr>
<tr><td>Depression</td><td>20002</td><td>(Interview) self-reported depression</td><td>If ‘x’== 1286</td><td>Not including post-natal depression</td></tr>
<tr><td>Asthma</td><td>t42014</td><td>(Algorithmically defined outcomes) Asthma from self-report or hospital admission EHR</td><td>If ‘x’ < assessment date</td><td>http://biobank.ctsu.ox.ac.uk/crystal/refer.cgi?id=4124</td></tr>
</table>

Chronic respiratory diseases (not including COPD)	20002	(Interview) self-reported bronchiectasis, interstitial lung disease, asbestosis, pulmonary fibrosis, fibrosing alveolitis/unspecified alveolitis, respiratory failure	if `x'==1114 | `x'==1115 | `x'==1120 | `x'==1121 | `x'==1122 | `x'==1124	Individuals reporting; other respiratory problems, sleep apnea, pleurisy, pneumothorax, spontaneous pneumothorax/recurrent pneumothorax, pleural plaques (not known asbestosis), pleural effusion not excluded as these are not chronic or not debilitating<br><br>Respiratory infection, pneumonia, lung abscess, empyema not included as individuals with these conditions are unlikely to attend the examination center
COPD	42016	(Algorithmically defined outcomes) COPD from self-report or hospital admission EHR	If ‘x’ < assessment date	http://biobank.ctsu.ox.ac.uk/crystal/refer.cgi?id=4125
Chronic immunological/ systemic diseases	20002	(Interview) self-reported rheumatoid arthritis, vasculitis, giant cell/temporal arteritis, polymyalgia rheumatica, Wegners granulmatosis, microscopic polyarteritis, polyartertis nodosa, systemic lupus erythematosis/sle, sjogren's syndrome/sicca syndrome, dermatopolymyositis, dermatomyositis, polymyositis, scleroderma/systemic sclerosis, chronic fatigue syndrome, antiphospholipid syndrome	if `x'==1464 | `x'==1372 | `x'==1376 | `x'==1377 | `x'==1378 | `x'==1379 | `x'==1380 | `x'==1381 | `x'==1382 | `x'==1383 | `x'==1480 | `x'==1481 | `x'==1384 | `x'==1482 | `x'==1564	Individuals reporting; sarcoidosis, connective tissue disorder, Raynaud’s phenomenon/disease not excluded<br><br>Allergy/hypersensitivity/anaphylaxis variables are not considered
Liver failure/cirrhosis	20002	(Interview) self-reported liver failure/cirrhosis, primary biliary cirrhosis, alcoholic liver disease / alcoholic cirrhosis	if `x'==1158 | `x'==1506 | `x'==1604	
CVD at baseline	42000, 42006, 42008, 42010, 42012	(Algorithmically defined outcomes) myocardial infarction, stroke, ischaemic stroke, subarachnoid haemorrhage, brain haemorrhage	ICD-10:<br>MI: I21, I22, I23, I24.1, I25.2<br>Subarachnoid haemorrhage: I60<br>Intracerebral haemorrhage: I61<br>Cerebral infarction: I63<br>Stroke, not specified as haemorrhage or Infarction: I64.X<br><br>ICD-9:<br>MI: 410, 411, 412.X, 429.79<br>Subarachnoid haemorrhage: 430.X<br>Intracerebral haemorrhage: 431.X<br>Occlusion of cerebral arteries: 434.X<br>Cerebral thrombosis: 434.0<br>Cerebral embolism: 434.1<br>Cerebral artery occlusion, unspecified: 434.9<br>Acute, but ill-defined, cerebrovascular disease: 436.X	http://biobank.ctsu.ox.ac.uk/crystal/refer.cgi?id=461<br>http://biobank.ctsu.ox.ac.uk/crystal/refer.cgi?id=462

	41270, 41280, 41271, 41281	(Hospital In-patient data) Angina, heart failure	ICD-10: Angina (I20): I20.0, I20.1, I20.8, I20.9 Heart failure (I11, I13, I50): I50.0, I50.1, I50.9, I11.0, I11.9, I13.0, I13.2, I13.9) ICD-9: Angina: 4139 Heart failure: 4280, 4281, 4289	http://biobank.ctsu.ox.ac.uk/crystal/field.cgi?id=41270
Cancer at baseline	2453	(Questionnaire) self-reported cancer	If ‘x’==1	http://biobank.ctsu.ox.ac.uk/crystal/coding.cgi?id=6 http://biobank.ctsu.ox.ac.uk/crystal/field.cgi?id=40006 http://biobank.ctsu.ox.ac.uk/crystal/field.cgi?id=40013&nl=1
	20001	(Interview) self-reported cancer excluding non-melanoma skin cancer	If ‘x’ != . & ‘x’ != 1060 | ‘x’ != 1061 | ‘x’ != 1062 | ‘x’ != 1073	
	40006	(Cancer Registry) Any cancer-type (C-D48) excluding non-melanoma skin cancers (ICD-10; C44, ICD-9; 173)		
Diabetes (excluding gestational diabetes)	2443	(Questionnaire) self-reported diabetes	If ‘x’==1 & n_4041_0_0 (gestational diabetes) !=1	
	6153 (women) 6177 (men	(Questionnaire) self-reported Insulin user	If ‘x’==3	
	20002	(Interview) self-reported diabetes	if ‘x’== 1220 | ‘x’== 1222 | ‘x’==1223	
	30750	(Biochemistry) HbA1c	If ‘x’ ≥48 mmol/l	
Hypertension	6150	(Questionnaire) self-reported High Blood Pressure	If ‘x’==4	
	6153 (women) 6177 (men	(Questionnaire) self-reported blood pressure medication	If ‘x’==2	
	4080, 4079, 93, 94	Measured (manual or automated) systolic and diastolic BP	If SBP≥140 | DBP≥90	
	20002	(Interview) self-reported hypertension, essential hypertension	if ‘x’==1065| ‘x’==1072	
Psychological/ psychiatric problems	20002	(Interview) self-reported schizophrenia, mania/bipolar disorder/manic depression, deliberate self-harm/suicide attempt, post-traumatic stress disorder	If ‘x’== 1289 | ‘x’== 1290 | ‘x’== 1291 | ‘x’== 1469	
Substance abuse/dependency	20002	(Interview) self-reported alcohol dependency, opioid dependency, other substance abuse/dependency	if `x'==1408 | `x'== 1408 | `x'== 1410	
Anorexia/bulimia/ other eating disorder	20002	(Interview) self-reported anorexia/bulimia/other eating disorder	if `x'==1470	
Chronic/degenerative neurological problem	20002	(Interview) self-reported chronic/degenerative neurological problem, Parkinson’s disease, dementia/Alzheimer’s/cognitive	if `x'==1258 | `x'==1259 | `x'==1260 | `x'==1261 | `x'==1262 | `x'==1263 | `x'== 1397	

		impairment, motor neuron disease, myasthenia gravis, multiple sclerosis, other demyelinating disease (not multiple sclerosis)		
Chronic widespread pain	2956	(Questionnaire)<br>"Have you had pains all over the body for more than 3 months?"	if `x'==1	doi: 10.1007/s11657-015-0252-1

Table S2. Age-sex specific cardiorespiratory fitness cut-offs in men and women.

W/kg fat-free mass						
	Women			Men		
	Low	Medium	High	Low	Medium	High
< 50yrs	< 2.71	2.71 - 3.83	> 3.83	< 3.40	3.40 - 4.49	> 4.49
50 - 59.9yrs	< 2.42	2.42 - 3.46	> 3.46	< 3.09	3.09 - 4.24	> 4.24
60 – 69.9yrs	< 1.90	1.90 - 2.90	> 2.90	< 2.56	2.56 - 3.78	> 3.78
≥ 70yrs	< 1.53	1.53 - 2.36	> 2.36	< 2.08	2.08 - 3.20	> 3.20
**W/kg body weight**						
	Women			Men		
	Low	Medium	High	Low	Medium	High
< 50yrs	< 1.73	1.73 - 2.50	> 2.50	< 2.54	2.54 - 3.43	> 3.43
50 - 59.9yrs	< 1.51	1.51 - 2.20	> 2.20	< 2.29	2.29 - 3.18	> 3.18
60 – 69.9yrs	< 1.18	1.18 - 1.82	> 1.82	< 1.87	1.87 - 2.81	> 2.81
≥ 70yrs	< 0.98	0.98 - 1.47	> 1.47	< 1.51	1.51 - 2.36	> 2.36

Table S3. Cross-tabulation of BMI- and body fat percentage categories in participants free from baseline CVD/cancer with ≥ 2 years of follow-up.

**BMI** \ **BF%***	Low	Medium	High	Total
Normal weight	18,350	5,214	87	23,651
Overweight	5,373	19,803	3,826	29,002
Obese	70	4,001	10,219	14,290
Total	23,793	29,018	14,132	

N=66,943 *Cut-offs based on the sex-specific distribution of the sample in BMI-categories. BF% ranges for women are; Low: 9.4 – 35.2, Medium: 35.3 – 41.9, High: 42.0 - 66.1. BF% ranges for men are; Low: 5.0 – 21.8, Medium: 28.9 – 29.2, High: 29.3 - 47.2.

Table S4. Cross-tabulation of cardiorespiratory fitness categories based on w/kg and w/fat-free mas

W/fat-free mass<br>W/kg	Low	Medium	High	Total
Low	13,576	2,005	0	15,851
Medium	2,084	25,412	3,378	30,874
High	1	3,481	27,232	30,714
Total	15,661	30,898	30,610	

N=77,169

Table S5. Sex-stratified cardiorespiratory fitness-fatness combinations and All-Cause Mortality

	**Model 1**<br>CRF/kg + BMI<br><br>All participants<br>(adjusting for prevalent cancer/CVD)		**Model 2**<br>CRF/kg + BMI<br><br>Free from CVD/cancer at baseline and removing early follow-up		**Model 4**<br>CRF/FFM + BF%<br><br>Free from CVD/cancer at baseline and removing early follow-up	
	Men	Women	Men	Women	Men	Women
N (deaths)	36,432 (1066)	40,737 (665)	31,863 (621)	35,080 (385)	31,863 (621)	35,080 (385)
Normal weight /unfit	1.98 (1.42, 2.77)	1.38 (0.97, 1.95)	1.98 (1.28, 3.05)	0.94 (0.59, 1.51)	1.71 (1.10, 2.67)	0.63 (0.41, 0.98)
n (deaths)	1609 (68)	2764 (49)	1398 (39)	2397 (24)	1674 (42)	3301 (29)
Normal weight /medium fit	1.66 (1.23, 2.25)	1.30 (0.99, 1.73)	1.62 (1.10, 2.40)	1.00 (0.69, 1.43)	1.37 (0.90, 2.07)	0.56 (0.38, 0.82)
n (deaths)	3273 (102)	6312 (103)	2908 (60)	5432 (53)	3218 (55)	6049 (44)
Normal weight /fit	*ref*	*ref*	*ref*	*ref*	*ref*	*ref*
n (deaths)	4666 (72)	8337 (95)	4197 (44)	7319 (65)	3639 (38)	5912 (69)
Overweight /unfit	1.71 (1.28, 2.30)	1.39 (0.99, 1.93)	1.84 (1.26, 2.69)	1.07 (0.69, 1.66)	1.63 (1.10, 2.41)	0.81 (0.53, 1.25)
n (deaths)	3218 (130)	2906 (60)	2770 (80)	2466 (32)	3016 (83)	2343 (32)
Overweight /medium fit	1.33 (1.02, 1.75)	1.11 (0.83, 1.48)	1.28 (0.90, 1.82)	0.87 (0.60, 1.27)	1.16 (0.80, 1.68)	0.71 (0.49, 1.02)
n (deaths)	7557 (202)	6330 (98)	6649 (114)	5409 (53)	6516 (112)	5138 (55)
Overweight /fit	1.33 (1.01, 1.75)	1.21 (0.91, 1.61)	1.36 (0.96, 1.93)	1.07 (0.75, 1.53)	1.15 (0.79, 1.67)	0.70 (0.49, 1.01)
n (deaths)	7593 (180)	5791 (96)	6725 (112)	4983 (58)	6720 (107)	5285 (56)
Obese /unfit	1.92 (1.42, 2.59)	1.64 (1.15, 2.31)	1.94 (1.31, 2.88)	1.51 (0.98, 2.32)	1.78 (1.17, 2.71)	1.09 (0.68, 1.77)
n (deaths)	2657 (128)	2507 (56)	2203 (71)	2151 (36)	1681 (63)	1370 (25)
Obese /medium fit	1.50 (1.11, 2.03)	1.36 (0.99, 1.88)	1.50 (1.02, 2.22)	1.09 (0.72, 1.65)	1.41 (0.95, 2.10)	0.87 (0.58, 1.30)
n (deaths)	3704 (120)	3722 (70)	3190 (69)	3193 (40)	3013 (79)	2847 (42)
Obese / fit	1.38 (0.98, 1.95)	1.20 (0.82, 1.77)	1.19 (0.75, 1.89)	1.13 (0.70, 1.83)	0.94 (0.60, 1.48)	0.68 (0.44, 1.05)
n (deaths)	2155 (64)	2068 (38)	1823 (32)	1730 (24)	2386 (42)	2835 (33)

Hazard ratios with 95% CI. Adjusted for age (time scale), Townsend index, education, partner status, ethnicity, employment status, diet pattern, alcohol intake, smoking status, TV-viewing, depression, asthma, hormone replacement therapy (women only), beta-blockers, calcium-channel blockers, statins, hypertension, diabetes (including CVD and cancer in model 1). Follow-up is commenced 2 years after baseline in models (2 and 4). CRF; cardiorespiratory fitness in watts, FFM; fat-free mass, BF%; body fat percent, BW; Body weight, CVD; cardiovascular disease,

Table S6. Cardiorespiratory fitness-fatness combinations and All-Cause Mortality among participants with a graded exercise test

	Model 1<br>CRF/kg + BMI<br><br>All participants<br>(adjusting for prevalent cancer/CVD)	Model 2<br>CRF/kg + BMI<br><br>Free from CVD/cancer at baseline and removing early follow-up	Model 3<br>CRF/kg + BMI<br><br>Never-smokers,<br>free from CVD/cancer,<br>removing early follow-up	Model 4<br>CRF/FFM + BF%<br><br>Free from CVD/cancer at baseline and removing early follow-up	Model 5<br>CRF/FFM + BF%<br><br>Never-smokers,<br>free from CVD/cancer,<br>removing early follow-up
N (deaths)	72,846 (1565)	63,539 (924)	36,829 (396)	63,539 (924)	36,829 (396)
Normal weight /unfit	1.74 (1.35, 2.23)	1.41 (1.01, 1.96)	1.39 (0.87, 2.23)	1.01 (0.73, 1.39)	0.97 (0.63, 1.52)
n (deaths)	3909 (104)	3409 (56)	2153 (28)	4502 (63)	2883 (33)
Normal weight /medium fit	1.47 (1.19, 1.83)	1.27 (0.96, 1.67)	0.97 (0.63, 1.48)	0.86 (0.64, 1.14)	0.67 (0.44, 1.01)
n (deaths)	9043 (184)	7873 (102)	4983 (39)	8871 (92)	5525 (38)
Normal weight /fit	*ref*	*ref*	*ref*	*ref*	*ref*
n (deaths)	12,466 (149)	11,086 (99)	6744 (48)	9224 (99)	5537 (51)
Overweight /unfit	1.64 (1.31, 2.06)	1.52 (1.14, 2.02)	1.47 (0.96, 2.24)	1.18 (0.89, 1.57)	0.98 (0.64, 1.50)
n (deaths)	5392 (167)	4635 (100)	2736 (44)	4752 (103)	2781 (43)
Overweight /medium fit	1.25 (1.02, 1.53)	1.08 (0.84, 1.40)	1.17 (0.81, 1.70)	0.87 (0.67, 1.12)	0.81 (0.56, 1.17)
n (deaths)	13,345 (279)	11,659 (159)	6657 (75)	11,237 (157)	6512 (72)
Overweight /fit	1.29 (1.05, 1.58)	1.22 (0.95, 1.57)	1.21 (0.84, 1.76)	0.88 (0.69, 1.14)	0.76 (0.52, 1.11)
n (deaths)	13,064 (264)	11,486 (167)	6268 (68)	11,766 (160)	6488 (62)
Obese /unfit	1.83 (1.44, 2.32)	1.73 (1.28, 2.34)	1.44 (0.90, 2.30)	1.29 (0.93, 1.78)	0.86 (0.51, 1.47)
n (deaths)	4370 (151)	3730 (92)	2158 (33)	2492 (69)	1401 (21)
Obese /medium fit	1.43 (1.14, 1.79	1.19 (0.89, 1.59)	1.12 (0.72, 1.75)	1.05 (0.79, 1.40)	0.91 (0.60, 1.40)
n (deaths)	7147 (174)	6184 (96)	3336 (36)	5594 (112)	3042 (43)
Obese /fit	1.26 (0.97, 1.65	1.11 (0.79, 1.56)	1.43 (0.87, 2.35)	0.73 (0.53, 1.01)	0.87 (0.55, 1.36)
n (deaths)	4110 (93)	3477 (53)	1794 (25)	5102 (69)	2660 (33)

Hazard ratios with 95% CI. Adjusted for age (time scale), sex, Townsend index, education, partner status, ethnicity, employment status, diet pattern, alcohol intake, smoking status, TV-viewing, depression, asthma, hormone replacement therapy (women only), beta-blockers, calcium-channel blockers, statins, hypertension and diabetes (including CVD and cancer in model 1). Follow-up is commenced 2 years after baseline in models (2-5). CRF; cardiorespiratory fitness in watts, BMI; body mass index, FFM; fat-free mass, BF%; body fat percent, CVD; cardiovascular disease

Table S7. Cardiorespiratory fitness-fatness combinations and all-cause Mortality, age-stratified

	**Model 1**<br>CRF/kg + BMI<br><br>All participants<br>(adjusting for prevalent cancer/CVD)		**Model 2**<br>CRF/kg + BMI<br><br>Free from CVD/cancer at baseline and removing early follow-up		**Model 4**<br>CRF/FFM + BF%<br><br>Free from CVD/cancer at baseline and removing early follow-up	
	<60 yo	≥60 yo	<60 yo	≥60 yo	<60 yo	≥60 yo
N (deaths)	40,680 (469)	36,489 (1262)	37,071 (295)	29,872 (711)	37,021 (295)	29,872 (711)
Normal weight /unfit	1.47 (0.94, 2.31)	1.70 (1.28, 2.26)	1.12 (0.62, 2.02)	1.48 (1.02, 2.15)	0.95 (0.58, 1.56)	1.03 (0.70, 1.51)
Normal weight /medium fit	1.56 (1.09, 2.24)	1.40 (1.09, 1.80)	1.37 (0.87, 2.16)	1.19 (0.86, 1.65)	0.78 (0.50, 1.22)	0.89 (0.63, 1.26)
Normal weight /fit	*ref*	*ref*	*ref*	*ref*	*ref*	*ref*
Overweight /unfit	1.80 (1.22, 2.64)	1.43 (1.11, 1.85)	1.64 (1.03, 2.63)	1.33 (0.95, 1.87)	1.05 (0.66, 1.65)	1.20 (0.85, 1.69)
Overweight /medium fit	0.94 (0.65, 1.37)	1.30 (1.04, 1.63)	0.74 (0.46, 1.19)	1.17 (0.87, 1.57)	0.71 (0.47, 1.09)	0.95 (0.70, 1.30)
Overweight /fit	1.16 (0.80, 1.66)	1.27 (1.00, 1.60)	1.10 (0.71, 1.71)	1.19 (0.89, 1.60)	0.68 (0.44, 1.05)	0.96 (0.70, 1.30)
Obese /unfit	1.72 (1.15, 2.56)	1.73 (1.33, 2.26)	1.51 (0.92, 2.48)	1.68 (1.19, 2.37)	1.07 (0.62, 1.83)	1.46 (1.01, 2.11)
Obese /medium fit	1.38 (0.93, 2.04)	1.40 (1.08, 1.81)	1.11 (0.68, 1.81)	1.31 (0.94, 1.83)	0.81 (0.50, 1.33)	1.18 (0.85, 1.65)
Obese /fit	1.01 (0.61, 1.68)	1.37 (1.02, 1.84)	0.94 (0.50, 1.77)	1.18 (0.80, 1.74)	0.49 (0.27, 0.91)	0.89 (0.62, 1.28)

Hazard ratios with 95% CI. Adjusted for age (time scale), Townsend index, education, partner status, ethnicity, employment status, diet pattern, alcohol intake, smoking status, TV-viewing, depression, asthma, hormone replacement therapy (women only), beta-blockers, calcium-channel blockers, statins, hypertension, diabetes (including CVD and cancer in model 1). Follow-up is commenced 2 years after baseline in models (2 and 4). CRF; cardiorespiratory fitness in watts, BMI; body mass index, FFM; fat-free mass, BF%; body fat percent, CVD; cardiovascular disease

Table S8. Cardiorespiratory fitness-fatness combinations and All-Cause Mortality using waist circumference as adiposity marker

	**Model 1** CRF/kg + waist circumference<br><br>All participants (adjusting for prevalent cancer/CVD)		**Model 2** CRF/kg + waist circumference<br><br>Free from CVD/cancer at baseline and removing early follow-up		**Model 4** CRF/FFM + waist circumference<br><br>Free from CVD/cancer at baseline and removing early follow-up	
	Men	Women	Men	Women	Men	Women
N (deaths)	36,427 (1065)	40,734 (665)	31,861 (621)	35,078 (385)	31,861 (621)	35,078 (385)
Normal weight /unfit	1.64 (1.34, 2.00)	1.21 (0.92, 1.59)	1.61 (1.24, 2.09)	0.89 (0.62, 1.29)	1.65 (1.28, 2.14)	0.77 (0.54, 1.10)
n (deaths)	4435 (138)	4682 (82)	3817 (107)	4035 (41)	4346 (119)	4857 (46)
Normal weight /medium fit	1.22 (1.02, 1.46)	1.11 (0.89, 1.39)	1.15 (0.91, 1.45)	0.85 (0.63, 1.14)	1.17 (0.92, 1.49)	0.73 (0.54, 0.99)
n (deaths)	9887 (260)	10,303 (158)	8761 (151)	8877 (79)	9016 (153)	9405 (78)
Normal weight /fit	*ref*	*ref*	*ref*	*ref*	*ref*	*ref*
n (deaths)	11,603 (216)	11,892 (152)	10,391 (138)	10,428 (101)	9607 (124)	9078 (97)
Obese /unfit	1.59 (1.28, 1.99)	1.48 (1.11, 1.97)	1.58 (1.18, 2.10)	1.37 (0.96, 1.97)	1.64 (1.21, 2.24)	1.29 (0.87, 1.91)
n (deaths)	3047 (143)	3496 (83)	2553 (83)	2980 (51)	2024 (69)	2158 (40)
Obese /medium fit	1.36 (1.10, 1.69)	1.15 (0.90, 1.49)	1.24 (0.95, 1.63)	1.03 (0.75, 1.43)	1.36 (1.03, 1.80)	0.98 (0.70, 1.36)
n (deaths)	4648 (164)	6063 (113)	3987 (92)	5160 (67)	3732 (93)	4631 (63)
Obese / fit	1.34 (1.05, 1.70)	1.04 (0.78, 1.37)	1.11 (0.80, 1.54)	0.98 (0.69, 1.40)	1.10 (0.81, 1.50)	0.89 (0.64, 1.24)
n (deaths)	2810 (99)	4304 (77)	2354 (50)	3603 (46)	3138 (63)	4954 (61)

Hazard ratios with 95% CI. Obesity was defined as waist circumference ≥88 cm for women and ≥102 cm for men. Adjusted for age (time scale), Townsend index, education, partner status, ethnicity, employment status, diet pattern, alcohol intake, smoking status, TV-viewing, depression, asthma, hormone replacement therapy (women only), beta-blockers, calcium-channel blockers, statins, hypertension, diabetes (including CVD and cancer in model 1). Follow-up is commenced 2 years after baseline in models (2 and 4). CRF; cardiorespiratory fitness in watts, FFM; fat-free mass, CVD; cardiovascular disease

Table S9. Cardiorespiratory fitness-fatness combinations and All-Cause Mortality omitting control for potential mediating variables

	Model 1<br>CRF/kg + BMI<br><br>All participants<br>(adjusting for prevalent cancer/CVD)	Model 2<br>CRF/kg + BMI<br><br>Free from CVD/cancer at baseline and removing early follow-up	Model 3<br>CRF/kg + BMI<br><br>Never-smokers,<br>free from CVD/cancer at baseline and removing early follow-up	Model 4<br>CRF/FFM + BF%<br><br>Free from CVD/cancer at baseline and removing early follow-up	Model 5<br>CRF/FFM + BF%<br><br>Never-smokers,<br>free from CVD/cancer at baseline and removing early follow-up
N (deaths)	71,169 (1731)	69,943 (1006)	38,884 (423)	69,943 (1006)	38,884 (423)
Normal weight /unfit	1.66 (1.31, 2.10)	1.41 (1.04, 1.93)	1.49 (0.95, 2.33)	1.04 (0.77, 1.41)	0.98 (0.64, 1.52)
Normal weight /medium fit	1.45 (1.18, 1.78)	1.27 (0.97, 1.65)	1.06 (0.71, 1.59)	0.86 (0.65, 1.12)	0.69 (0.46, 1.04)
Normal weight /fit	*ref*	*ref*	*ref*	*ref*	*ref*
Overweight /unfit	1.59 (1.29, 1.96)	1.56 (1.19, 2.04)	1.57 (1.05, 2.36)	1.24 (0.94, 1.62)	1.13 (0.76, 1.68)
Overweight /medium fit	1.22 (1.01, 1.48)	1.09 (0.85, 1.39)	1.22 (0.85, 1.75)	0.90 (0.70, 1.15)	0.89 (0.62, 1.28)
Overweight /fit	1.26 (1.04, 1.53)	1.21 (0.95, 1.54)	1.28 (0.89, 1.84)	0.89 (0.70, 1.15)	0.80 (0.55, 1.16)
Obese /unfit	1.90 (1.53, 2.35)	1.92 (1.46, 2.53)	1.70 (1.10, 2.62)	1.54 (1.15, 2.06)	1.14 (0.71, 1.82)
Obese /medium fit	1.48 (1.20, 1.83)	1.40 (1.07, 1.83)	1.37 (0.90, 2.09)	1.17 (0.90, 1.53)	1.04 (0.69, 1.56)
Obese /fit	1.38 (1.07, 1.76)	1.25 (0.90, 1.73)	1.64 (1.02, 2.65)	0.85 (0.63, 1.15)	1.05 (0.68, 1.61)

Hazard ratios with 95% CI. Adjusted for age (time scale), sex, Townsend index, education, partner status, ethnicity, employment status, diet pattern, alcohol intake, smoking status, TV-viewing, depression, asthma and hormone replacement therapy (women only and including CVD and cancer in model 1. Follow-up is commenced 2 years after baseline in models (2 and 4). CRF; cardiorespiratory fitness in watts, BMI; body mass index, FFM; fat-free mass, BF%; body fat percentage, CVD; cardiovascular disease.

Table S10. Cardiorespiratory fitness-fatness combinations and CVD- and cancer-specific mortality

	**Analysis 1** CRF/kg + BMI<br><br>All participants (adjusting for prevalent cancer/CVD)		**Analysis 4** CRF/FFM + BF%<br><br>Free from CVD/cancer at baseline and removing early follow-up	
	Cancer mortality	CVD mortality	Cancer mortality	CVD mortality
N (deaths)	71,169 (1121)	71,169 (327)	66943 (622)	66,953 (192)
Normal weight /unfit	1.47 (1.10, 1.96)	2.60 (1.31, 5.17)	0.81 (0.55, 1.20)	1.28 (0.59, 2.78)
n (deaths)	4373 (74)	4373 (19)	4975 (39)	4975 (13)
Normal weight /medium fit	1.33 (1.04, 1.69)	2.39 (1.28, 4.46)	0.77 (0.55, 1.08)	0.89 (0.42, 1.87)
n (deaths)	9585 (138)	9585 (31)	9267 (63)	9267 (14)
Normal weight /fit	*ref*	*ref*	*ref*	*ref*
n (deaths)	13,003 (124)	13,003 (15)	9551 (75)	9551 (14)
Overweight /unfit	1.38 (1.06, 1.80)	2.82 (1.54, 5.16)	0.99 (0.70, 1.40)	1.42 (0.71, 2.86)
n (deaths)	6124 (114)	6124 (40)	5359 (64)	5359 (22)
Overweight /medium fit	1.22 (0.97, 1.52)	1.80 (1.00, 3.24)	0.86 (0.64, 1.16)	1.11 (0.58, 2.13)
n (deaths)	13,887 (210)	13,887 (47)	11,654 (110)	11,654 (31)
Overweight /fit	1.19 (0.94, 1.50)	2.24 (1.26, 3.98)	0.79 (0.58, 1.07)	1.39 (0.75, 2.60)
n (deaths)	13,384 (186)	13,384 (53)	12,005 (100)	12,005 (38)
Obese /unfit	1.46 (1.11, 1.93)	3.71 (2.03, 6.76)	1.03 (0.69, 1.53)	2.28 (1.13, 4.58)
n (deaths)	5164 (98)	5165 (48)	3051 (42)	3051 (25)
Obese /medium fit	1.33 (1.03, 1.72)	2.75 (1.51, 5.00)	1.11 (0.80, 1.53)	1.22 (0.60, 2.49)
n (deaths)	7426 (120)	7426 (43)	5860 (81)	5860 (21)
Obese /fit	1.06 (0.77, 1.45)	3.27 (1.75, 6.10)	0.76 (0.52, 1.09)	0.95 (0.44, 2.06)
n (deaths)	4223 (57)	4223 (31)	5221 (48)	5221 (14)

Hazard ratios with 95% CI. Adjusted for age (time scale), Townsend index, education, partner status, ethnicity, employment status, diet pattern, alcohol intake, smoking status, TV-viewing, depression, asthma, hormone replacement therapy (women only), beta-blockers, calcium-channel blockers, statins, hypertension, diabetes (including CVD and cancer in model 1). Follow-up is commenced 2 years after baseline in model 5. CVD mortality are ICD-10 codes I05-I89.9. Cancer mortality are ICD-10 codes C-D48. CRF; cardiorespiratory fitness in watts, BMI; body mass index, FFM; fat-free mass, BF%; body fat percentage, CVD; cardiovascular disease.

Figure S1. Participant Flowchart.

Participants attending baseline assessment, N=502 536

Participants offered cardiorespiratory fitness assessment,

n = 78 959 (baseline)

n = 20 208 (repeat)

N = 96 208

Participants with submaximal test (graded or constant resistance),

n = 68 665 (baseline)

n = 18 216 (repeat)

N = 84 406

Participants with CRF test passing quality control procedures,

n = 65 913 (baseline)

n = 17 768 (repeat)

N = 81 377

Participants without a history of respiratory disease (including COPD), chronic immunological or systemic disease, liver failure/cirrhosis, schizophrenia, mania/bipolar disorder/manic depression, deliberate self-harm/suicide attempt, post-traumatic stress disorder, substance abuse/dependency, eating disorders, chronic/degenerative neurological problems, chronic widespread pain, BMI<18.5 and measured BMI and %body fat,

N = 62 498 (baseline)

N = 16 845 (repeat)

N = 77 169

Keeping only baseline file in individuals with data both time-points,

N = 62 498 (baseline)

N = 14 671 (repeat)

N = 77 169

Imputed data on co-variates

N = 58 600 complete, 3 898 with variables imputed (baseline)

N = 14 267 complete, 404 with variables imputed (repeat)

N = 72 876 complete, 4 302 with variables imputed

Participants with no history of cancer or CVD

N = 54 637 (baseline)

N = 12 502 (repeat)

N = 67 139

Participants surviving >2 years after CRF assessment

N = 54 488 (baseline)

N = 12 455 (repeat)

N = 66 943

n = observations, N = unique participants, ‘baseline’ refers to the study baseline assessment, ‘repeat’ refers to individuals invited to repeat baseline measures (now including cardiorespiratory fitness assessment)
